# Proline metabolism supports metastasis formation and could be inhibited to selectively target metastasizing cancer cells

**DOI:** 10.1038/ncomms15267

**Published:** 2017-05-11

**Authors:** Ilaria Elia, Dorien Broekaert, Stefan Christen, Ruben Boon, Enrico Radaelli, Martin F. Orth, Catherine Verfaillie, Thomas G. P. Grünewald, Sarah-Maria Fendt

**Affiliations:** 1 Laboratory of Cellular Metabolism and Metabolic Regulation, VIB Center for Cancer Biology, VIB, Herestraat 49, Leuven 3000, Belgium; 2 Laboratory of Cellular Metabolism and Metabolic Regulation, Department of Oncology, KU Leuven and Leuven Cancer Institute (LKI), Herestraat 49, Leuven 3000, Belgium; 3 Stem Cell Institute, KU Leuven, Herestraat 49, Leuven 3000, Belgium; 4 Center for the Biology of Disease, VIB Leuven and Center for Human Genetics, KU Leuven, Herestraat 49, Leuven 3000, Belgium; 5 Max-Eder Research Group for Pediatric Sarcoma Biology, Institute of Pathology, LMU Munich, Thalkirchner Strasse 36, Munich 80337, Germany

## Abstract

Metastases are the leading cause of mortality in patients with cancer. Metastasis formation requires cancer cells to adapt their cellular phenotype. However, how metabolism supports this adaptation of cancer cells is poorly defined. We use 2D versus 3D cultivation to induce a shift in the cellular phenotype of breast cancer cells. We discover that proline catabolism via proline dehydrogenase (Prodh) supports growth of breast cancer cells in 3D culture. Subsequently, we link proline catabolism to *in vivo* metastasis formation. In particular, we find that *PRODH* expression and proline catabolism is increased in metastases compared to primary breast cancers of patients and mice. Moreover, inhibiting Prodh is sufficient to impair formation of lung metastases in the orthotopic 4T1 and EMT6.5 mouse models, without adverse effects on healthy tissue and organ function. In conclusion, we discover that Prodh is a potential drug target for inhibiting metastasis formation.

## Abstract (web summary)

Metastasizing cancer cells rewire their metabolism to support their malignant phenotypes. Here, the authors show that the acquisition of a metastatic phenotype in breast cancer cell lines results in increased proline catabolism and that inhibition of this pathway decreases lung metastasis formation in two mouse models.

Metabolic reprogramming is recognized as a hallmark of cancer cells that supports cancer growth^1^. Investigating how metabolism supports cancer growth resulted in several metabolism-based drugs that are now in clinical trial^2^. Yet, some cancer cells within a progressing tumour acquire additional cellular phenotypes, such as motility, invasion, survival and colonization capacity, which are supporting cancer progression towards metastasis formation^3^. Thus, identifying how metabolism supports shifts in the cancer cell phenotype that can contribute to metastasis formation has the potential to identify innovative drug targets against cancer progression.

Ninety percent of all cancer deaths are caused by metastases in distant organs^4^. The metastasis formation cascade consists of several stages^5^,^6^: First, cancer cells disseminating from the primary tumour invade the surrounding tissue and intravasate into the circulation. Next, cancer cells infiltrate and colonize a distant organ. At this stage, cancer cells can remain dormant or in a stable micrometastasis state for weeks to years, but eventually they will grow and form macrometastases, which results in established secondary tumours. Several studies have focussed on metabolic reprogramming during the early steps of metastasis formation, when cancer cells disseminate from the primary tumour, invade the surrounding tissue and survive in the circulation^7^,^8^,^9^. From a clinical perspective, however, the later steps in metastasis formation are of specific interest, because patients often present to the clinics when cancer cells have already infiltrated a distant organ^10^,^11^. Yet, our knowledge on how metabolism supports cancer cell survival and colonization of a distant organ is limited to a few studies^7^,^12^,^13^.

Here we address the questions how metabolism supports phenotypic shifts in breast cancer cells and to which extent inhibiting these changes in metabolism can counteract *in vivo* lung metastasis formation. In particular, we enforce a phenotypic shift in breast cancer cells by cultivating them either as monolayer in two-dimensional (2D) culture or as spheroids in three-dimensional (3D) culture. Next, we identify differences in the cellular metabolism of cells cultured in 2D versus 3D conditions. Finally, we investigate whether inhibiting the identified metabolic differences impairs *in vivo* metastasis formation without adverse effect on healthy tissue and organ function in mice. We discover that breast cancer cells grown in spheroids (3D) compared to attached monolayers (2D) increase proline catabolism via the enzyme proline dehydrogenase (Prodh). Inhibiting Prodh activity results in impaired spheroidal growth and in a dose-dependent decrease in lung metastasis formation in two mouse models. Pharmacological Prodh inhibition has no adverse effects *in vitro* on non-transformed mammary epithelial cells and *in vivo* on healthy tissue and organ function of mice. Thus we identify Prodh as promising drug target against breast cancer-derived metastasis formation.

## Results

### Proline catabolism distinguishes 2D from 3D growth

We used transformed human MCF10A H-Ras^V12^ mammary epithelial cells to study phenotypic shifts induced by 2D versus 3D cultivation. This cell line has been used to study gene expression as well as signalling pathway activity during spheroidal growth^14^,^15^. MCF10A H-Ras^V12^ cells were derived from immortalized and non-tumorigenic breast epithelial cell line MCF10A (ref. 16). These cells were transduced with the oncogenic driver H-Ras^V12^, which is of relevance to the human breast cancer situation, since 50% of the human breast cancers display increased H-Ras activity^17^. To achieve spheroidal growth, we cultured MCF10A H-Ras^V12^ cells on soft-agar coated plates in DMEM-F12 media (Supplementary Fig. 1). To compare metabolism during spheroidal and attached monolayer growth, we applied ^13^C tracer analysis^18^. Since this method has so far not been used in soft-agar cultures, we tested the suitability of four different metabolite extraction methods^19^,^20^,^21^,^22^. We evaluated metabolite recovery, cellular energy charge and cellular protein recovery resulting from the four tested methods. We found that methanol–chloroform extraction combined with a mechanical disruption of the spheroids is well suited to extract metabolites from spheroids cultured on soft-agar (Supplementary Fig. 2). Consequently, we performed all our subsequent experiments using this method.

**Figure 1:**
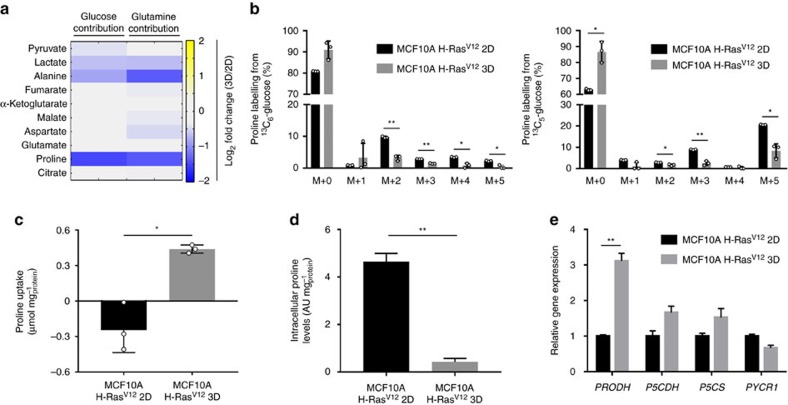
Proline catabolism distinguishes 3D from 2D growth. (**a**) Total contribution of ^13^C_6_-glucose and ^13^C_5_-glutamine to different organic acids and amino acids in MCF10A H-Ras^V12^ spheroids (3D) versus attached cells (2D). Changes in total contribution >25% with a *P* value≤0.05 are depicted. (**b**) Proline labelling from ^13^C_6_-glucose and ^13^C_5_-glutamine in MCF10A H-Ras^V12^ spheroids (3D) versus attached cells (2D). (**c**) Proline uptake in MCF10A H-Ras^V12^ spheroids (3D) versus attached cells (2D). (**d**) Intracellular levels of proline in MCF10A H-Ras^V12^ spheroids (3D) versus attached cells (2D). (**e**) Relative expression of proline catabolism (*PRODH* and *P5CDH*) and biosynthesis (*P5CS* and *PYCR1*) genes in MCF10A H-Ras^V12^ spheroids (3D) versus attached cells (2D) measured by qRT–PCR. The number of biological replicates for each experiment was *n*≥3. All error bars represent s.d. Two-tailed unpaired Student's *T*-test was performed. *P≤0.05; **P≤0.01.

Cancer cells growing either in attached monolayers (2D) or spheroids (3D) consume substantial amounts of the nutrients glucose and glutamine^2^,^23^,^24^. Yet, it remains elusive whether these nutrients are used to fuel metabolite production similarly in both culture conditions. Thus, we tested the contribution of both nutrients to organic and amino acid production in MCF10A H-Ras^V12^ in both conditions using ^13^C_6_-glucose and ^13^C_5_-glutamine. Interestingly, the most pronounced change observed was a decrease in the ^13^C contribution from both nutrients to the production of the amino acid proline in MCF10A H-Ras^V12^ (Fig. 1a,b and Supplementary Fig. 3), which indicates that these cells switch to a different proline metabolism when grown in spheroids.

To validate whether proline catabolism or biosynthesis are different in MCF10A H-Ras^V12^ cells during spheroidal growth, we quantified proline uptake, intracellular proline levels and the expression of *PRODH* and pyrroline-5-carboxylate dehydrogenase (*P5CDH*) (proline catabolism), as well as pyrroline-5-carboxylate synthase (*P5CS*) and pyrroline-5-carboxylate reductase 1 (*PYCR1*) (proline biosynthesis) in both growth conditions. We found that cells consumed proline from the media only during spheroidal growth, while the same cells secreted proline to the media during attached monolayer growth (Fig. 1c). In accordance, intracellular proline levels were lower in cells grown in spheroids than the same cells grown in monolayer (Fig. 1d). This suggested that cells grown in spheroids require more proline, because spheroidal growth might result in proline catabolism. Indeed, the expression of the proline catabolism enzyme *PRODH* was significantly increased by approximately 300% in MCF10A H-Ras^V12^ cells during spheroidal growth (*P*≤0.01, two-tailed unpaired Student's *T*-test) (Fig. 1e), while the expression level of *P5CDH* and that of the proline biosynthesis genes *P5CS* and *PYCR1* remained unchanged (Fig. 1e). Together, these data provide evidence that proline catabolism distinguishes spheroidal (3D) from attached (2D) growth in MCF10A H-Ras^V12^ cells.

### Proline catabolism via Prodh supports 3D growth

**Figure 2:**
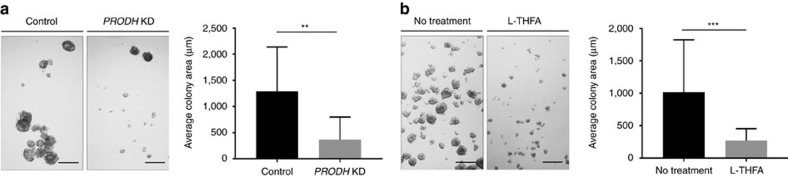
Proline catabolism via Prodh supports 3D growth. (**a**) Representative pictures and size quantification of MCF10A H-Ras^V12^ spheroids transduced with an inducible lentiviral CRISPR with or without guide for *PRODH*. CRISPR expression was induced with 0.1 μg ml^−1^ doxycycline. Analysis was performed 5 days after *PRODH* knockdown (KD) induction. (**b**) Representative pictures and size quantification of MCF10A H-Ras^V12^ spheroids with or without L-THFA treatment. Treatment was started at day 0. Analysis was performed at day 5 of treatment. Scale bar: 150 μm. The number of biological replicates for each experiment was *n*≥3. All error bars represent s.d. Two-tailed unpaired Student's *T*-test was performed. **P≤0.01; ***P≤0.001.

To investigate whether proline catabolism is required for spheroidal growth of MCF10A H-Ras^V12^ cells, we employed a genetic and catalytic inhibition of Prodh. To this end, we generated a doxycyclineinducible CRISPR interference knockdown of *PRODH* in MCF10A H-Ras^V12^ cells, which had an efficiency of 96% (Supplementary Fig. 4a). Strikingly, knockdown of *PRODH* impaired spheroidal growth of MCF10A H-Ras^V12^ by approximately 70% based on spheroid size (Fig. 2a).

We next tested whether inhibition of Prodh activity could elicit a similar effect on spheroidal growth by using the compound L-tetrahydro-2-furoic acid (L-THFA), which was reported to inhibit Prodh activity in 2D cultures^25^. Since increased Prodh activity results in decreased intracellular levels of proline (Fig. 1d), we argued that L-THFA treatment should increase intracellular proline levels. Consistently, we found that L-THFA treatment increased intracellular proline levels in MCF10A H-Ras^V12^ spheroids (Supplementary Fig. 4b) indicating that the compound effectively inhibits Prodh activity. Similar to the *PRODH* knockdown, L-THFA treatment reduced the spheroid size of MCF10A H-Ras^V12^ spheroid growth by approximately 75% (Fig. 2b). Collectively, these data show that Prodh activity is required for optimal spheroidal growth of MCF10A H-Ras^V12^ cells and suggested that Prodh may serve as a drug target.

### Prodh activity depends on P5C recycling during 3D growth

**Figure 3:**
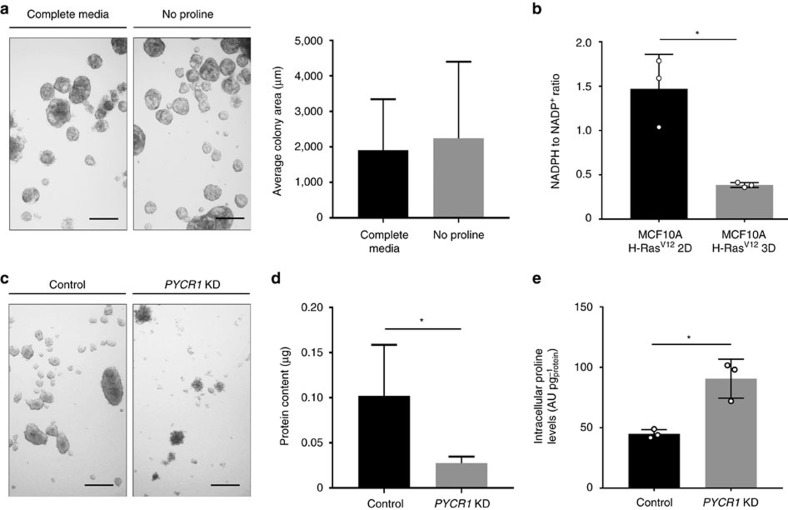
Prodh activity depends on P5C recycling via Pycr1. (**a**) Representative pictures and size quantification of MCF10A H-Ras^V12^ spheroids in complete media and media without proline. Analysis was performed at day 5. Scale bar: 150 μm. (**b**) NADPH to NADP^+^ ratio in MCF10A H-Ras^V12^ spheroids (3D) versus attached (2D) cells. (**c**) Representative pictures of MCF10A H-Ras^V12^ spheroids transduced with a lentiviral vector with shRNA for either *PYCR1* or a scrambled sequence. Analysis was performed at day 5. Scale bar: 150 μm. (**d**) Protein content in MCF10A H-Ras^V12^ spheroids transduced with a lentiviral vector with shRNA for either *PYCR1* (KD) or a scrambled sequence. Analysis was performed at day 5. (**e**) Intracellular levels of proline in MCF10A H-Ras^V12^ spheroids transduced with a lentiviral vector with shRNA for either *PYCR1* (KD) or scrambled sequence. Analysis was performed at day 5. Total ion counts were normalized for protein content. The number of biological replicates for each experiment was *n*≥3. All error bars represent s.d. Two-tailed unpaired Student's *T*-test was performed. *P≤0.05.

Subsequently, we investigated whether proline withdrawal from the media can impair spheroidal growth. Surprisingly, we found that proline removal from the media had no effect on the growth of MCF10A H-Ras^V12^ spheroids (Fig. 3a).

Interestingly, Prodh activity yields the metabolite pyrroline 5-carboxylic acid (P5C), which can be recycled to proline via the NADPH consuming proline biosynthesis enzyme Pycr^26^. Thus, we hypothesized that P5C recycling to proline via Pycr sustains Prodh activity. Consistently, we found that the NADPH to NADP^+^ ratio was decreased during spheroidal growth (Fig. 3b).

To further confirm the compensatory role of P5C recycling, we knocked down enzymes involved in proline recycling (*PYCR1*, *PYCR2*, *PYCR3*) (Supplementary Fig. 4c). Removal of proline from the media in combination with knockdown of *PYCR1* strongly impaired spheroidal growth of MCF10A H-Ras^V12^ (Supplementary Fig. 4d). This implied that *PYCR1* is the predominant *PYCRx* isoform generating proline from P5C in MCF10A H-Ras^V12^ spheroids. In line with the hypothesis that P5C recycling is important for sustaining Prodh activity, we found that *PYCR1* knockdown in MCF10A H-Ras^V12^ cells impaired spheroid growth by 73%, despite proline availability in the media (Fig. 3c,d). In agreement with a decreased Prodh activity in *PYCR1* knockdown cells, we observed that proline levels increased compared to control spheroids (Fig. 3e). Notably, while spheroidal growth of *PYCR1* knockdown cells was decreased, the remaining spheroids additionally showed increased aggregation, which indicates an additional function of Pycr1 that is independent of sustaining Prodh activity. This observation is in line with the finding that Pycr is involved in nucleotide and endoplasmic reticulum homeostasis^26^,^27^. Taken together, our data show that Prodh activity during spheroidal growth is sustained by P5C recycling via Pycr1.

### Prodh activity generates ATP during 3D growth

**Figure 4:**
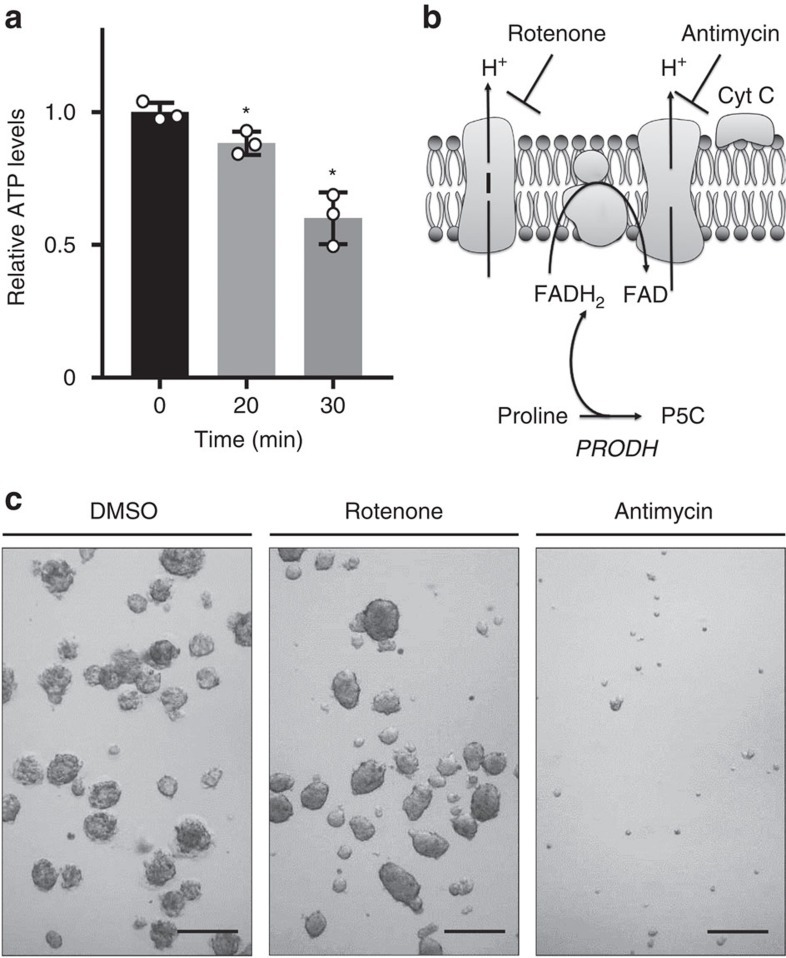
Prodh activity generates ATP during 3D growth. (**a**) Time-resolved ATP levels of MCF10A H-Ras^V12^ spheroids upon treatment with the Prodh inhibitor L-THFA. (**b**) Schematic representation of Rotenone and Antimycin A mode of action as well as ATP generation from proline catabolism. (**c**) Representative pictures of MCF10A H-Ras^V12^ spheroids upon treatment with either rotenone or antimycin A. Treatment was started at day 0. Analysis was performed at day 5 of treatment. Scale bar: 150 μm. The number of biological replicates for each experiment was *n*≥3. All error bars represent s.d. Two-tailed unpaired Student's *T*-test was performed. *P≤0.05.

It was shown previously that Prodh activity can contribute to mitochondrial energy production by feeding electrons into the electron transport chain^28^. To investigate whether proline catabolism via Prodh supports ATP production in spheroids, we performed a dynamic metabolomics experiment in MCF10A H-Ras^V12^ spheroids upon acute inhibition of Prodh activity using L-THFA. Interestingly, inhibition of Prodh activity by L-THFA significantly decreased ATP levels in MCF10A H-Ras^V12^ spheroids (*P*≤0.05, two-tailed unpaired Student's *T*-test) (Fig. 4a). Because ATP production via Prodh requires the activity of complex III of the electron transport chain, but not that of complex I (Fig. 4b), we hypothesized that spheroidal growth of MCF10A H-Ras^V12^ is more susceptible to inhibition of complex III than of complex I. In agreement, we found that complex III inhibitor antimycin A, but not the complex I inhibitor rotenone, impaired spheroidal growth of MCF10A H-Ras^V12^ cells (Fig. 4c). Collectively, our data show that Prodh activity supports spheroidal growth by sustaining ATP production.

### Prodh activity is specifically relevant in transformed cells

Our data suggest that Prodh may constitute a promising drug target against metastasis formation, especially if its activity would be specifically relevant for growth of transformed cells, but not of normal cells.

**Figure 5:**
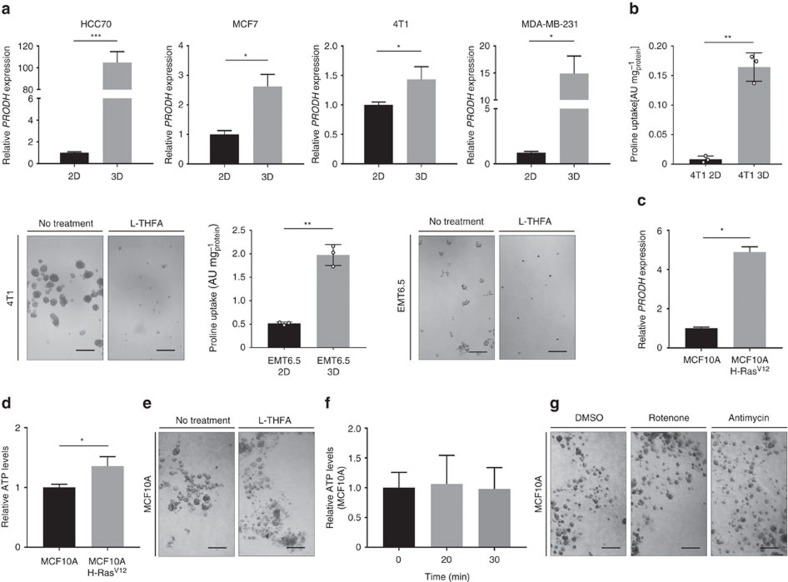
Prodh activity is specifically relevant in transformed cells. (**a**) Relative expression of *PRODH* in HCC70, MCF7, 4T1 and MDA-MB-231 spheroids (3D) versus the corresponding attached (2D) cells. (**b**) Proline uptake of 4T1 and EMT6.5 spheroids versus attached cells and representative pictures of 4T1 and EMT6.5 spheroids with or without L-THFA treatment. Treatment was started at day 0. Analysis was performed 5 days after treatment. Scale bar: 150 μm (4T1 spheroids) and 75 μm (EMT6.5 spheroids). (**c**) Relative expression of *PRODH* in MCF10A H-Ras^V12^ spheroids compared to MCF10A acini. (**d**) Relative ATP levels of MCF10A H-Ras^V12^ spheroids compared to MCF10A acini. (**e**) Representative pictures of MCF10A acini with or without L-THFA treatment. Treatment was started at day 0. Analysis was performed 5 days after treatment. Scale bar: 150 μm. (**f**) Time-resolved ATP levels of MCF10A acini upon treatment with the Prodh inhibitor L-THFA. (**g**) Representative pictures of MCF10A acini upon treatment with either Rotenone or Antimycin A. Treatment was started at day 0. Analysis was performed 5 days after treatment. Scale bar: 150 μm. To each experiment, 0.5% Matrigel was added to the media of both cell lines to allow acini formation of non-transformed MCF10A cells and to enable the comparison between MCF10A H-Ras^V12^ and MCF10A cells. The number of biological replicates for each experiment was *n*≥3. All error bars represent s.d. Two-tailed unpaired Student's *T*-test was performed. *P≤0.05; **P≤0.01; ***P≤0.001.

Thus, we tested whether *PRODH* expression was increased in transformed breast cancer cells during spheroidal growth. We measured the expression of *PRODH* in cell lines isolated from human or murine breast cancers (MCF7, HCC70, MDA-MB-231 and 4T1) during spheroidal (3D) compared to attached (2D) growth. We found that in all tested breast cancer cell lines *PRODH* expression increased during spheroidal growth (Fig. 5a). Moreover, we confirmed that 4T1 and EMT6.5 breast cancer cell lines, which we used for subsequent *in vivo* studies, increased proline uptake and were impaired by L-THFA treatment during spheroidal growth (Fig. 5b). This suggests that Prodh is relevant for spheroidal growth in breast cancer cell lines beyond MCF10A H-Ras^V12^.

Next, we tested whether Prodh activity is required for acinus formation of non-transformed MCF10A cells, which is a normal, non-invasive form of spheroidal growth^29^. Since non-transformed MCF10A cells fail to form acini without matrix attachment, we added 0.5% Matrigel to the DMEM-F12 media. We ruled out that Matrigel addition significantly changed organic and amino acid metabolism as well as growth upon Prodh inhibition in MCF10A H-Ras^V12^ spheroids (*P*>0.05, two-tailed unpaired Student's *T*-test; Supplementary Fig. 5). Interestingly, we found that *PRODH* expression and ATP levels were significantly lower in MCF10A acini compared to MCF10A H-Ras^V12^ spheroids (*P*≤0.05, two-tailed unpaired Student's *T*-test; Fig. 5c,d), suggesting that Prodh activity is not required in non-transformed cells. In support of this notion, Prodh inhibition by L-THFA did neither impair acini formation nor reduce ATP levels of MCF10A cells (Fig. 5e,f). Similarly, inhibition of complex III or complex I had no effect on acini formation (Fig. 5g). Together, these data show that Prodh activity in general and ATP production via Prodh in particular are not required during normal acini formation of non-transformed mammary epithelial cells.

### Prodh activity is increased in metastases tissue

**Figure 6:**
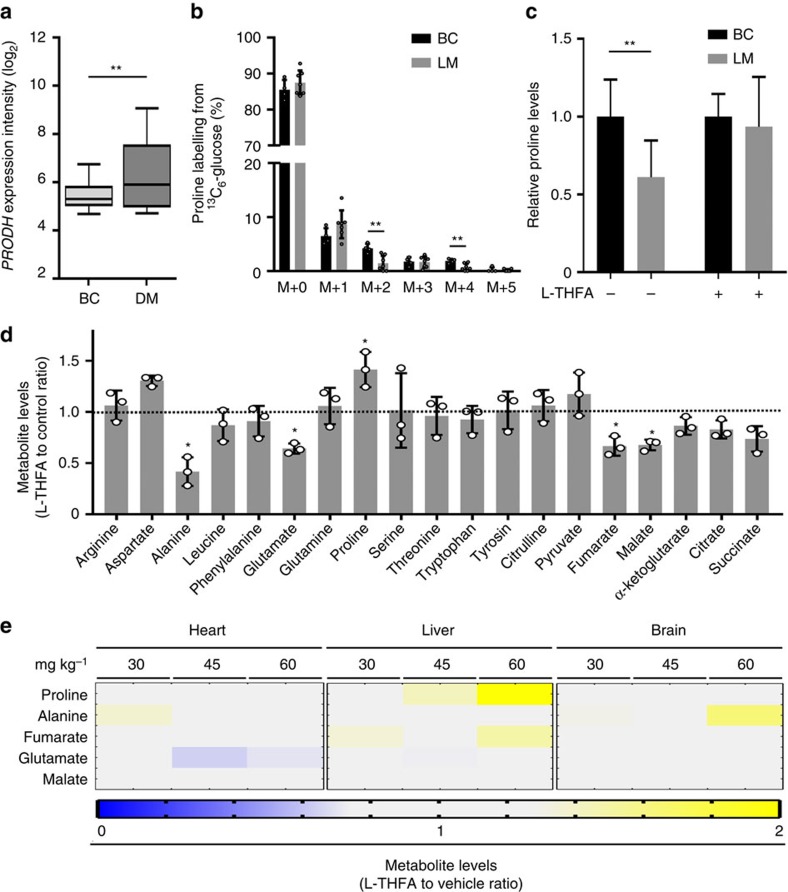
Prodh activity is specifically relevant in metastasis tissue. (**a**) *PRODH* expression levels in primary breast cancer (BC) tissue (GSE20711) and breast cancer-derived metastases (DM) tissue (GSE14017) from patients. Data are shown as medians (bar) with the 25th–75th percentile range (box) and the 10th–90th percentile range (whiskers). Two-tailed unpaired Student's *T*-test with Welch's correction was performed. **P≤0.01. (**b**) Proline labelling from ^13^C_6_-glucose in primary breast cancers (BC) and the resulting lung metastases (LM) of BALB/c mice orthotopically injected with murine 4T1 breast cancer cells. Four mice with primary BC and resulting LM (*n*=7) were analysed. Two-tailed Student's *T*-test with *F*-testing to confirm equal variance was performed. **P≤0.01. (**c**) Intracellular proline levels in primary breast cancers (BC) and the resulting lung metastases (LM) of BALB/c mice orthotopically injected with murine 4T1 breast cancer cells and treated with either L-THFA or vehicle normalized to the respective control condition. For the vehicle-treated group, *n*=8 mice with primary BC and resulting LM (*n*=11) were analysed. For the L-THFA-treated group, *n*=5 mice with primary breast cancer and resulting LM (*n*=5) were analysed. Two-tailed Student's *T*-test with *F*-testing to confirm equal variance was performed. **P≤0.01. (**d**) Relative metabolite levels of MCF10A H-Ras^V12^ spheroids treated with or without L-THFA. Two-tailed unpaired Student's *T*-test was performed. *P≤0.05. (**e**) Relative metabolite levels of the heart, liver and brain of mice treated with different doses of L-THFA compared to vehicle (PBS). Changes in metabolite levels >27% are depicted (*n*≥5). Two-tailed Student's *T*-test with *F*-testing to confirm equal variance was performed. Error bars represent s.d. unless otherwise noted.

Our *in vitro* data suggest that Prodh may be a drug target against metastasis formation, which is supported by the fact that *PRODH* is significantly higher expressed in metastases compared to primary breast cancers in patients (*P*≤0.01, two-tailed unpaired Student's *T*-test with Welchs correction; Fig. 6a). To test whether proline catabolism is indeed relevant for metastasis formation, we used an orthotopic 4T1 breast cancer mouse model. This model allowed us to investigate primary tumours and lung metastases within the same animal. As readout for Prodh activity, we measured enrichment of proline from ^13^C_6_-glucose and proline levels in 4T1 primary breast cancers and the corresponding lung metastases using *in vivo*^13^C tracer analysis. This analysis revealed that both parameters were reduced in lung metastases as compared to the primary breast cancer tumours (Fig. 6b,c), which is consistent with increased proline catabolism in metastases tissue.

We next assessed potential adverse effects of the Prodh inhibitor L-THFA in mice. Since L-THFA was, to our knowledge, never applied *in vivo*, we measured alterations in metabolite levels in MCF10A H-Ras^V12^ spheroids upon treatment with L-THFA to define which metabolic changes need to be monitored *in vivo* to exclude adverse effects on healthy tissue. We found that mainly proline, alanine, glutamate, fumarate and malate levels were significantly changed upon treatment with L-THFA (*P*≤0.05, two-tailed unpaired Student's *T*-test; Fig. 6d). Since Prodh is physiologically expressed at high levels in the brain, heart and liver (human protein atlas), we measured the levels of the five metabolites in the brain, heart and liver of BALB/c mice treated with increasing doses of L-THFA (30, 45, 60 mg kg^−1^). The compound was administered daily via intraperitoneal injections and metabolite levels were measured at day 16 of treatment. We found that only the lowest tested dose of L-THFA treatment did not result in a substantial increase in proline levels in these organs (Fig. 6e). Moreover, at this dose only minor and non-significant changes in alanine, glutamate, fumarate and malate levels were observed (*P*>0.05, two-tailed Student's *T*-test with *F*-testing to confirm equal variance).

To investigate the adverse effects beyond the level of central metabolism, we performed a full plasma/blood analysis and histopathology on BALB/c mice injected for 16 days with 30 mg kg^−1^ L-THFA. This analysis showed that L-THFA treatment at this dose did not result in significant changes in haematology, clinical chemistry and organ histopathology compared to vehicle treatment of BALB/c mice (*P*>0.05, two-tailed Student's *T*-test with *F*-testing to confirm equal variance; Supplementary Fig. 6). Based on this analysis, we decided to treat mice for all consecutive experiments with an L-THFA dose of ≤30 mg kg^−1^, which allowed safe administration for at least 16 days without obvious adverse effects.

### Prodh inhibition impairs lung metastases formation

Finally, we tested whether inhibition of Prodh activity is sufficient to impair *in vivo* metastasis formation in the 4T1 orthotopic breast cancer mouse model^30^. We induced primary tumour growth by injection of 4T1 breast cancer cells to the mammary fat pad of 6-week-old BALB/c mice. After 4 days when the primary tumour nodule was established and palpable, we started daily intraperitoneal injections of L-THFA at ≤30 mg kg^−1^. Sixteen days after treatment, we analysed proline levels (to confirm Prodh inhibition in the metastases) and the number of metastases in the lungs as well as the primary breast tumour weight.

We found that proline levels were lower in the metastasis tissue compared to the primary breast cancer tissue only in animals treated with vehicle (Fig. 6c). Upon L-THFA treatment, this difference was not present anymore (Fig. 6c), which showed that L-THFA treatment resulted in the expected inhibition of Prodh activity within the metastasis tissue.

**Figure 7:**
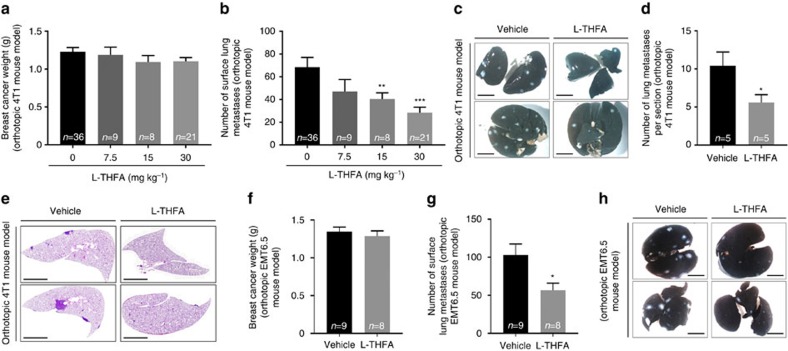
Prodh inhibition impairs lung metastases formation. (**a**) Primary tumour weight, (**b**) the number of surface lung metastases and (**c**) representative pictures of ink-stained lungs with white tissue indicating metastases of BALB/c mice orthotopically injected with murine 4T1 breast cancer cells treated with vehicle or L-THFA at different concentrations for 16 days: 0 mg kg^−1^ (*n*=36, 4 independent cohorts), 7.5 mg kg^−1^ (*n*=9), 15 mg kg^−1^ (*n*=8), and 30 mg kg^−1^ (*n*=21, 3 independent cohorts). Treatment was started after primary breast tumours were formed. Scale bar: 0.5 cm. Two-tailed unpaired Student's *T*-test with Welch's correction was performed. **P≤0.01; ***P≤0.001. (**d**) Number of lung metastases per lung section and (**e**) representative pictures of H&E-stained lung sections of BALB/c mice orthotopically injected with murine 4T1 breast cancer cells treated with vehicle or L-THFA at 30 mg kg^−1^ (*n*=5 per condition). Scale bar: 2.7 mm. Two-tailed Student's *T*-test with *F*-testing to confirm equal variance was performed. *P≤0.05. (**f**) Primary tumour weight, (**g**) the number of surface lung metastases and (**h**) representative pictures of ink-stained lungs (black) with metastases (white) of BALB/c mice orthotopically injected with murine EMT6.5 breast cancer cells treated with vehicle (*n*=9) or L-THFA at 15 mg kg^−1^ (*n*=8) for 18 days. Scale bar: 0.5 cm. Two-tailed Student's *T*-test with *F*-testing to confirm equal variance was performed. *P≤0.05. All error bars represent s.e.m.

Strikingly, the weight of the primary breast cancer tumours remained unchanged upon L-THFA treatment (Fig. 7a), whereas the number of surface lung metastases decreased in a dose-dependent manner, with the strongest reduction in metastases (∼60%) at 30 mg kg^−1^ (Fig. 7b,c). Next, we performed haematoxylin and eosin (H&E) staining of the lungs from animals treated with this dose, which confirmed the significantly reduced total number of metastases per lung section by approximately 50% (*P*≤0.05, two-tailed Student's *T*-test with Welch's correction) (Fig. 7d,e). Finally, we validated the effect of Prodh inhibition on metastasis formation in the orthotopic EMT6.5 breast cancer mouse model^30^,^31^ (Fig. 7f–h).

In summary, our data demonstrate that Prodh inhibition is sufficient to impair formation of breast cancer-derived lung metastases.

## Discussion

Here we show that proline catabolism via Prodh distinguishes breast cancer cells growing in spheroids (3D) from those growing in attached (2D) conditions. Inhibiting Prodh activity specifically impairs the capacity of breast cancer cells to form spheroids *in vitro* and lung metastases *in vivo*. Treatment with the Prodh inhibitor L-THFA had no adverse effects on non-transformed cells *in vitro* and *in vivo*. Thus, our data suggest Prodh as a promising drug target for preventing lung metastasis formation in breast cancer.

Unlike many other non-essential amino acids, the catabolism and biosynthesis of proline is catalysed by two different enzymes, namely Prodh and Pycr, that are coupled to two different co-factors. Thus, various roles of proline metabolism in transformed and non-transformed cells are possible. Indeed, inhibition of proline biosynthesis has been linked to unresolved endoplasmic reticulum stress in yeast and in cancer cells^27^,^32^. Moreover, it has been shown that proline biosynthesis can contribute to sustaining intracellular nucleotide levels^26^. In certain cancer cells, proline biosynthesis inhibition was found to impair tumorigenic potential and to decrease attached monolayer growth^26^,^27^. Yet, inhibition of proline catabolism in attached monolayer cultures resulted across different *in vitro* studies in both anti- and pro-growth/survival effects^28^,^33^,^34^,^35^,^36^,^37^,^38^,^39^. Moreover, the *in vivo* role of Prodh activity in cancers has so far not been studied. This is challenging for advancing Prodh as a drug target, since it remains unclear under which *in vivo* conditions Prodh can be targeted to counteract cancer. We discovered that Prodh activity specifically impairs *in vitro* spheroidal growth and *in vivo* metastasis formation of breast cancer cells. Moreover, we show that treatment with the Prodh inhibitor L-THFA does not result in any adverse effects in normal cells and organ function (evaluated by organ metabolomics, haematology, clinical chemistry and organ histopathology) in mice. Thus, our study highlights Prodh as promising and likely safe therapeutic target for preventing breast cancer-derived metastasis formation.

In contrast to our recent finding that pyruvate carboxylase-dependent anaplerosis distinguishes the metabolism of primary breast cancers from that of the resulting lung metastases due to the nutrient microenvironment^13^,^40^,^41^, the data presented here indicate that the dependence of lung metastases on Prodh activity is driven by shifts in the cellular phenotype. In accordance, proline catabolism seems to be specifically important during early stages of metastatic growth but less relevant in established secondary tumours. Consistent with this notion, we found that *PRODH* expression inversely correlated with spheroid size (Supplementary Fig. 7a) and that L-THFA treatment started after spheroid formation did not impair spheroid growth any longer (Supplementary Fig. 7b). Also, these data are consistent with the recent finding that MYC, which negatively regulates *PRODH* expression^34^, shows lower expression in micrometastases (defined by signatures of quiescent and dormancy genes) than macrometastases^42^. Consistently, we found that *MYC* expression is inversely correlated with *PRODH* expression in primary breast cancer tissue compared to metastases tissue of patients (Fig. 6a and Supplementary Fig. 7c).

The ability of cancer cells to perform phenotypic shifts is only one aspect of metastasis formation, and multiple other factors, including the extracellular matrix composition and stiffness, are important additional factors for metastasis formation^43^,^44^. Hence, it is tempting to speculate that the need of breast cancer cells for proline catabolism may depend on specific site/organ of metastasis. However, the finding that MYC, which negatively regulates *PRODH* expression, clusters with metastasis size rather than with the metastatic site^42^, suggests that Prodh inhibition may be independent of the organ in which metastases form.

Our data suggest that targeting Prodh activity could have the potential to be effective against dormant breast cancer cells and micrometastases. Notably, targeting such cancer cells is of specific interest for breast cancer treatment, because infiltration of distant organs leading to micrometastases formation is a very early event that often occurs before the primary tumour is resected^10^,^11^ and which results years later in established secondary tumours and 90% of breast cancer deaths^2^. In summary, we identify Prodh as promising drug target against breast cancer-derived metastasis formation.

## Methods

### Cell culture

We generated MCF10A cells expressing H-Ras^V12^ (MCF10A H-Ras^V12^) as well as control cells expressing an empty pLA vector (MCF10A) (Supplementary Fig. 1a). MCF10A and MCF10A H-Ras^V12^ cells were grown in DMEM-F12 supplemented with 5% horse serum, 1% penicillin (50 units ml^−1^), 1% streptomycin (50 μg ml^−1^), 0.5 μg ml^−1^ hydrocortisone, 100 ng ml^−1^ cholera toxin, 10 μg ml^−1^ insulin and 20 ng ml^−1^ recombinant human EGF. MCF7, HCC70 and MDA-MB-231 cells were cultured in DMEM supplemented with 10% fetal bovine serum, 1% penicillin (50 units ml^−1^), 1% streptomycin (50 μg ml^−1^) and 0.15 mM proline. 4T1 cells were cultured in Roswell Park Memorial Institute medium with pyruvate supplement, 10% fetal bovine serum, 1% penicillin (50 units ml^−1^) and 1% streptomycin (50 μg ml^−1^). All cell lines were confirmed to be mycoplasma-free by the Mycoalert Detection Kit (Lonza).

MCF10A, MCF7, HCC70, MDA-MB-231 and 4T1 cell lines were purchased from ATCC. All human cell lines were validated by DNA fingerprinting. The EMT6.5 cell line is a single-cell clone from the EMT6 cell line available from ATCC^31^ and kindly provided by Professor Robin Anderson (Peter MacCallum Cancer Centre). None of the cell lines used for this study are listed in the database of commonly misidentified cell lines maintained by ICLAC. Both the 4T1 and EMT6 cell line-based orthotopic mouse models are widely used and accepted models for metastasis research^30^. Primary breast cancer of these models spontaneously metastasize within a short time frame and with a 100% penetrance.

Two different 3D growth conditions were tested (Supplementary Fig. 1): For soft-agar culture, a base layer of agar was prepared in six-well plates. Soft-agar was dissolved at 1% in sterile distilled water in a microwave oven and was mixed with prewarmed (37 °C) medium at a ratio of 1:1. The base layer of agar mixture was transferred into a six-well plate at 3 ml per well. Soft-agar was left to solidify at room temperature. Reduced growth factor Matrigel (0.5%) without phenol red was added into the cold DMEM-F12 media before the seeding by using tips precooled to −20 °C. MCF10A cells were plated on top of the base agar at 15,000 cells per well. For ultra-low attachment, cells were suspended sparsely and plated at 15,000 cells per well in six-well ultra-low Corning attachment plates. Unless otherwise noted, MCF10A H-Ras^V12^ were cultured on soft-agar without Matrigel addition. For ^13^C carbon incorporation from glucose (Cambridge Isotope Laboratories, 99%) and glutamine (Sigma, 98%), cells were incubated for 5 days with labelled substrates. All analyses were performed after 5 days of incubation at 37 °C in a 5% CO_2_ incubator.

L-THFA was purchased from Sigma-Aldrich and used at the concentration of 5 mM (MCF10A, MCF10A H-Ras^V12^ and 4T1 cells) and 10 mM (EMT6.5 cells). The compound was added at day 0 and pictures were taken at day 5. For ATP analysis, L-THFA was used at day 5 for 0, 20 and 30 min at the concentration of 10 mM. Rotenone and Antimycin A were purchased from Sigma-Aldrich and used at the concentration of 5 and 2 μM, respectively. The compounds were added at day 0 and pictures were taken at day 5. All growth experiments were performed in *n*≥3 biological replicates.

### shRNA knockdowns

The lentiviral pLKO sh*PYCR1* (NM_153824.1-398s1c1), sh*PYCR2* (NM_013328.2-996s1c1), sh*PYCR3* (NM_023078.x-1629s1c1) and scrambled control were purchased from the Broad Institute (Supplementary Table 1).

### CRISPR interference knockdowns

The lentiviral CRISPR interference plasmid pHR-SFFV-KRAB-dCas9-P2A-mCherry was obtained from AddGene (Plasmid #60954). Inducibility was achieved by subcloning the KRAB-dCas9-P2A-mcherry behind the TetO promoter of the lentiviral pTripZ vector (Open Biosystems RHS4750) through Gibson cloning. The U6 promoter and tracer sequence were cloned from pX330-U6-Chimeric-BB-CBh-hSpCas9, obtained from AddGene (Plasmid #42230) through Gibson cloning. Guide specific for *PRODH* was selected by using the online guide design website available from http://crispr.mit.edu. From these, the top guide (GGCCCTGTAGTGCCGAAGCA) was selected and cloned 3′ to the U6 promoter through Gibson cloning^45^,^46^. Lentiviral particles were produced in HEK293 cells. MCF10A and MCF10A H-Ras^V12^ cells were selected with puromycin 1 μg ml^−1^. The expression of the CRISPR was induced with doxycycline 0.1 μg ml^−1^. As a control, an inducible CRISPR line lacking the guide for PRODH was generated.

### Flow cytometry

In brief, MCF10A cells and MCF10A-derived colonies were lysed in trypsin 0.05% and 1 × 10^6^ single cells were resuspended and incubated with antibodies at concentrations of 0.2 μg per 100 μl for CD24 conjugated with phycoerythrin (PE) (12-0247-42) and 0.1 μg per 100 μl for CD44 conjugated with PE-cyanine7 (Cy7) (560533), both purchased from BD Biosciences. Samples were stained in ice in the dark for 15 min, and then the cells were washed twice with cold PBS, resuspended in 400 μl PBS and analysed using a flow cytometer (BD FACS Verse). As negative controls, cells were treated with either isotype-matched control antibodies or with no primary antibody.

### RNA isolation and quantitative real-time PCR

Total RNA was isolated with the PureLink RNA Mini Kit (Life Technologies). RNA (400 ng) was reverse transcribed into cDNA using a High-Capacity cDNA Reverse Transcription Kit (Life Technologies). The relative levels of gene transcripts compared to the control gene RPL-19 were determined by quantitative real-time PCR using SYBER Green PCR Master Mix (Life Technologies) and specific primers on a 7,500 Fast Real Time PCR System (Applied Biosystems, Life Technologies) (Supplementary Table 2). Amplification was performed at 95 °C for 10 min, followed by 40 cycles of 15 s at 95 °C and 1 min at 60 °C. The fold change in gene expression was calculated as:


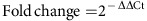


### Quenching and metabolite extraction methods

The quenching procedure applied was adapted from Sellick *et al*.^47^. Fifty-ml falcon tubes with methanol 60% and 10 mM ammonium acetate were precooled in a dry ice-ethanol bath (−40 °C) for 20 min. Colonies were transferred with a 1,000 μl pipette from the soft-agar plates to 15 ml falcon tubes and pelleted by centrifugation (1,400 r.p.m., 1 min, room temperature) to remove excess of medium. In this step, four wells of one six-well plate were pooled into one falcon tube (one replicate). Medium was aspirated by using a vacuum line and the remaining cell pellet was immediately added to the 50 ml falcon tube containing 5 ml of the quenching solution at −40 °C. Cells were pelleted by centrifugation (2,000 r.p.m., 30 s, room temperature) and the supernatant was removed by quickly inverting the tube. The cell pellet was washed a second time with quenching solution (−40 °C). After the second wash, the cell pellet was stored at −80 °C.

Four different extractions methods were tested. All the extraction procedures were performed in a dry ice-ethanol bath (−40 °C). The protocols were adapted from the established published methods. The details of each procedure are described separately below. The methanol/chloroform extraction procedure was adapted from de Koning *et al*.^19^. The samples were resuspended in 800 μl of precooled 60% methanol, before 500 μl of precooled chloroform were added. Samples were vortexed for 10 min at 4 °C and then centrifuged (max. speed, 10 min, 4 °C). The upper methanol/water phase, the intermediate protein layer and the lower chloroform phase were collected separately.

The hot ethanol extraction procedure was adapted from Bent *et al*.^20^. Ethanol 75% was preheated in a water bath at 80 °C for 20 min. Hot ethanol (1 ml) was poured over the cell pellet. The samples were immediately vortexed and then placed in the water bath for 3 min. After the 80 °C incubation, the samples were placed back in the cold bath (−40 °C) and then centrifuged (max. speed, 5 min, 4 °C). The supernatants and the proteins were collected separately.

The trichloroacetic acid extraction procedure was adapted from Tunnicliffe *et al*.^22^. Samples were resuspended in precooled 5% trichloroacetic acid and then centrifuged (max. speed, 5 min, 4 °C). The supernatants and the protein were collected separately.

The Acetonitrile–Methanol Extraction procedure was adapted from Rabinowitz *et al*.^21^. Each sample was resuspended in 1 ml of precooled acetonitrile/methanol/water (40:40:20) and centrifuged (max. speed, 5 min, 4 °C). The supernatants and the protein were collected separately.

By using a tissue lyser, a mechanical disruption step was performed after resuspension of the cell pellet with the chemicals of the respective extraction method at −40 °C for 5 min.

The extracts were dried using a vacuum concentrator. Fatty acids were dried at 20 °C for 1 h, while polar metabolites were dried at 4 °C over night. The samples were stored at −80 °C.

### Gas chromatography–mass spectrometric analysis

Metabolites were derivatized and measured as described before^48^. In brief, polar metabolites were derivatized with 20 mg ml^−1^ methoxyamine in pyridine for 90 min at 37 °C and subsequently with *N*-(tert-butyldimethylsilyl)-*N*-methyl-trifluoroacetamide, with 1% tert-butyldimethylchlorosilane for 60 min at 60 °C. Fatty acids were esterified with sulfuric acid/methanol for 72 h at 60 °C and then extracted with hexane. Metabolites were measured with a 7890A GC system (Agilent Technologies) combined with a 5975C Inert MS system (Agilent Technologies). One microlitre of samples was injected in splitless mode with an inlet temperature of 270 °C onto a DB35MS column. The carrier gas was helium with a flow rate of 1 ml min^−1^. For the measurement of polar metabolites, the GC oven was held at 100 °C for 3 min and then ramped to 300 °C with a gradient of 2.5 °C min^−1^. For the measurement of fatty acids, the oven was held at 80 °C for 1 min and ramped with 5 °C min^−1^ to 300 °C. The MS system was operated under electron impact ionization at 70 eV and a mass range of 100–650 a.m.u. was scanned.

Mass distribution vectors were extracted from the raw ion chromatograms using a custom Matlab M-file, which applies consistent integration bounds and baseline correction to each ion. Moreover, we corrected for naturally occurring isotopes using the method of Fernandez *et al*.^49^. Total contribution of carbon was calculated using the following equation^18^:


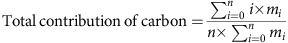


Here *n* is the number of C atoms in the metabolite, *i* the different mass isotopomers and *m* the abundance of a certain mass. For metabolite levels, arbitrary units of the metabolites of interest were normalized to an internal standard and protein content (*in vitro* experiments) or tissue weight (*in vivo* experiments). *In vivo*, the ^13^C enrichment of glucose in the blood of the mice was evaluated and confirmed to be similar between the infused animals.

### Liquid chromatography–mass spectrometric analysis

Polar metabolites were resuspended in 60% acetonitrile. Metabolites were measured using a Dionex UtiMate 3,000 LC System (Thermo Scientific) combined with a Q Exactive Orbitrap mass spectrometer (Thermo Scientific) and run in negative mode, using mass spectrometry based on accurate mass. Samples were injected onto a SeQuant ZIC/pHILIC Polymeric column (Merck Millipore)^50^. The solvent, composed of acetonitrile and ammonium acetate (pH=9.3, 10 mM), was used at a flow rate of 0.100 ml min^−1^. Data analysis was performed with the Xcalibur software. Metabolite levels were normalized to a fully ^13^C-labelled yeast extract and protein content. Alternatively, targeted measurements of polar metabolites were performed with a 1,290 Infinity II HPLC (Agilent) coupled to a 6,470 triple quadrupole mass spectrometer (Agilent). Samples were injected onto a iHILIC-Fusion(P) column with the above-mentioned solvents. Data analysis was performed with the Agilent Mass Hunter software.

### Analysis of gene expression microarray data

Microarray data of primary breast cancers (GSE20711)^51^ and breast cancer metastases (GSE14017)^52^ generated on Affymetrix HG-U133Plus2.0 chips were retrieved from the Gene Expression Omnibus and normalized simultaneously by Robust Multichip Average^53^ using custom brain array chip description files (v18, ENTREZG) yielding one optimized probe-set per gene^54^. Statistical significance of differences in the gene expression levels were analysed with a two-tailed unpaired Student's *T*-test with Welch's correction using the GraphPad Prism 5 software (GraphPad Software Inc.).

### *In vivo*^13^C tracer infusions

Six-week-old female BALB/c mice were orthotopically inoculated with one million 4T1 cells. Primary tumour growth and body weight was monitored every second day. One week after tumour initiation, surgical catherization of the jugular vein was carried out as follows: BALB/c mice were anesthetized with 3% isoflurane with 2% oxygen and a catheter was placed into the right jugular vein. A small incision between shoulder blades was made and the catheter was exteriorized at the back of the mouse and connected to an antenna part, which enables conscious handling of the mouse during infusion. The tubing was flushed with heparinized saline directly after the surgery and 3 days later. Animals were individually housed after surgery, and painkillers were administered (Carprofen, 5 mg kg^−1^ body weight subcutaneously) during surgery and 1 day there after^13^,^55^,^56^.

Two weeks after surgery, ^13^C_6_-labelled glucose (99% enriched; Sigma-Aldrich) infusions were performed (*n*=4). Mice were subjected to a continuous infusion of 0.03 mg ^13^C-labelled glucose per gram body weight per min for the course of 6 h with a glucose solution of 500 mg per ml ^13^C-labelled glucose. Subsequently, mice were killed using nembutal, blood was collected and the breast tumours and lung metastases were placed in cold saline, dissected in <3 min and immediately frozen using a liquid nitrogen cooled Biosqueezer (Biospec Products). The tissue was weighed (10–15 mg) and pulverized (Cryomill, Retsch) under liquid nitrogen conditions. The pulverized tissue was extracted for GC-MS and LC-MS analysis as described above. Additionally, blood enrichment of ^13^C glucose was analysed to ensure that no significant differences in blood enrichment per animal are observed. The animal study complies with ethical regulation and was approved by the KU Leuven ethics committee.

### *In vivo* drug treatment

Six-week-old female BALB/c mice were orthotopically inoculated with one million of either 4T1 or EMT6.5 cells. For inhibition of Prodh activity, L-THFA treatment was started after 4 days when the primary tumour nodule was established and palpable. Different doses of L-THFA (60 mg kg^−1^ (*n*=6), 45 mg kg^−1^ (*n*=6), 30 mg kg^−1^ (*n*=21 with 3 independent cohorts), 15 mg kg^−1^ (*n*=8), 7.5 mg kg^−1^ (*n*=9)) were injected daily intraperitoneally to healthy or cancer-bearing mice. L-THFA was dissolved in PBS and pH neutralized with NaOH. PBS was injected as a vehicle to control animals (*n*=36 with 4 independent cohorts). L-THFA-treated and control mice were randomly chosen. Mice were killed after 16 days (4T1 model) or 18 days (EMT6.5 model) of treatment. In case of cancer-bearing mice, lung metastases were visualized by intratracheal injection of ink solution (15% of final volume of black ink, water, 2–3 drops ammonia). Black ink-injected lungs were washed in distaining solution (100 ml 70% EtOH, 100 ml 37% formaldehyde, 5 ml glacial acetic acid) and then placed in fresh distaining solution overnight. Two different researchers in a blind manner counted white tumour nodules against a black lung background. The animal study complies with ethical regulation and was approved by the KU Leuven ethics committee.

### *In vivo* histopathology analysis

A short-term toxicology study was carried out in order to rule out potential adverse effect/toxicity of the drug under the same treatment conditions used for the original efficacy study. Briefly, six-week-old BALB/c females were randomly divided into two groups (*n*=5 per group) and treated with 30 mg kg^−1^ of L-THFA (group I) or vehicle only (group II) once a day for 16 days via peritoneal injection. During the treatment period, the following clinical, physiological and behavioural parameters were constantly monitored and recorded: body weight, food and water consumption, body condition score, home cage activity, and locomotion. Twenty-four hours after the last injection, animals were killed by CO_2_ asphyxiation. Terminal blood collection was also performed via cardiac puncture. Next, the mice underwent complete necropsy with gross examination and organ weights. The following organs/tissues were finally sampled and immersion fixed for 48–72 h in 10% neutral buffered formalin: truncal skin, entire head, spine, salivary glands, esophagus, larynx, trachea, lungs, heart, gastrointestinal tract, liver and gall bladder, pancreas, kidneys, ovaries, uterus, urinary bladder, lymph nodes (cervical, mesenteric), spleen, thymus, left hindlimb, and sternum.

Formalin-fixed tissues samples were routinely processed and embedded in paraffin blocks for histopathological examination (Thermo Scientific Excelsior AS Tissue Processor and HistoStar Embedding Workstation). 5 μm thick sections obtained from these blocks (Thermo Scientific Microm HM355S microtome) were then stained with H&E (Leica ST5010 Autostainer XL) and evaluated under a Leica DM 2,500 light microscope by a board-certified veterinary pathologist. Trimming and orientation of the various organs/tissues collected were performed following the RITA guidelines (revised guides for organ sampling and trimming in rats and mice). Microscopic lesions observed during histopathological examination were classified according to the INHAND (International Harmonization of Nomenclature and Diagnostic Criteria) system. Histopathological examination was performed in a blind manner and details concerning experimental design and tested compounds were revealed only at the end of the study. Whole-blood samples were collected in BD Microtainer K2E tubes (ref. #365955). Haematology was performed using the scil Vet abc Plus analyser. Clinical chemistry was conducted on plasma separated from the whole blood.

For the quantification of pulmonary metastasis, dissected lung samples were gently infused via the trachea with 10% neutral buffered formalin and then processed for histopathological examination as previously described. 5 μm thick sections obtained from the resulting paraffin blocks were stained with H&E. Reconstruction and absolute counts of metastatic lesions were finally performed on four step sections with an interval of 30 μm and therefore spanning an overall parenchymal thickness of 110 μm. The animal study complies with ethical regulation and was approved by the KU Leuven ethics committee.

### Statistical analysis

Two-tailed unpaired Student's *T*-tests were performed on *n*≥3 biological replicates. For the *in vivo* data, statistical significance was calculated by standard two-tailed Student's *T*-tests with *F*-testing to confirm equal variance. In case of unequal variance, Welch's correction was performed. Detection of mathematical outliers was performed using Grubb's test in GraphPad. Sample size for all experiments was chosen empirically. Independent experiments were pooled and analysed together whenever possible as detailed in figure legends. All graphs show mean±s.d. or s.e.m. as indicated in the figure legends.

### Data availability

Microarray data of primary breast cancers (GSE20711)^51^ and breast cancer metastases (GSE14017)^52^ generated on Affymetrix HG-U133Plus2.0 chips are available through Gene Expression Omnibus. The authors declare that all the other data supporting the findings of this study are available within the article and its Supplementary Information files and from the corresponding author upon reasonable request.

## Additional information

**How to cite this article:** Elia, I. *et al*. Proline metabolism supports metastasis formation and could be inhibited to selectively target metastasizing cancer cells. *Nat. Commun.* **8**, 15267 doi: 10.1038/ncomms15267 (2017).

**Publisher's note**: Springer Nature remains neutral with regard to jurisdictional claims in published maps and institutional affiliations.

## Supplementary Material

Supplementary Information Supplementary Figures, Supplementary Tables and Supplementary References
