# Supplementary material for: Proline metabolism supports metastasis formation and could be inhibited to selectively target metastasizing cancer cells
Source: Nat Commun. 2017 May 11;8:15267. doi: 10.1038/ncomms15267 (PMC5437289; doi:10.1038/ncomms15267)
Supplement: Supplementary Information — Supplementary Figures, Supplementary Tables and Supplementary References [file ncomms15267-s1.pdf]

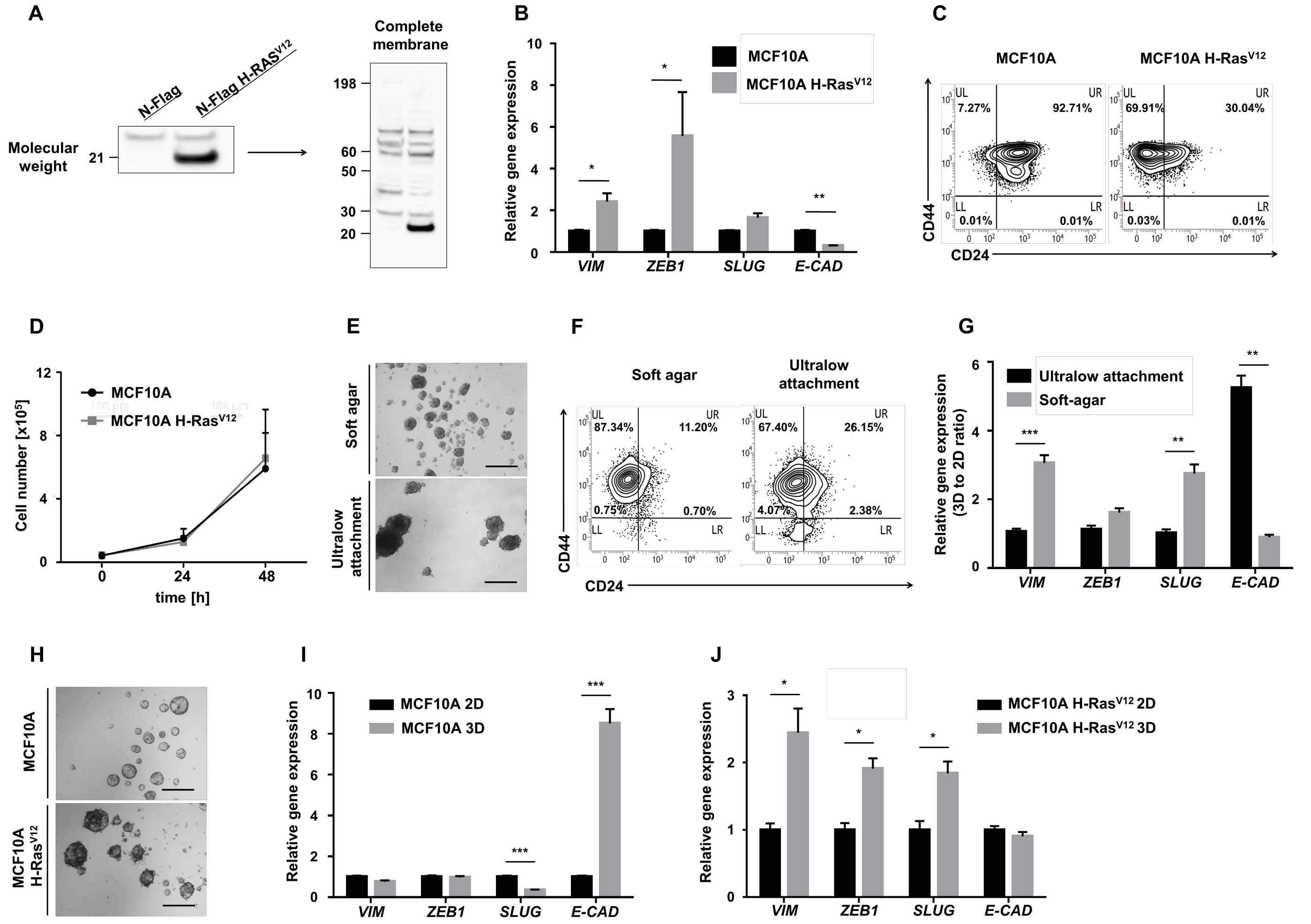

Supplementary Figure 1: Soft-agar cultures are a suitable 3D cultivation system to study spheroidal growth

(A) Western blot analysis for N-Flag in MCF10A cells overexpressing N-Flag H-Ras^{V12} compared to control MCF10A cells. MCF10A cells were infected with either a Flag-pLA vector carrying the H-Ras^{V12} sequence (MCF10A H-Ras^{V12}) or an empty pLA vector (MCF10A). (B) Expression of mesenchymal markers *VIM, ZEB1, SLUG* and epithelial marker *E-CAD* in cells cultured in attached (2D) conditions. (C) FACS profiles for CD44^{high}/CD24$_{low}$ markers of MCF10A and MCF10A H-Ras^{V12} cells cultured in 2D. (D) Proliferation of MCF10A H-Ras^{V12} and MCF10A cells cultured in attached (2D) conditions. (E) Representative pictures of MCF10A H-Ras^{V12} cells cultured on soft agar or ultralow attachment plates. Analysis was performed at day 5. Scale bar: 150 µm. (F) FACS profiles for CD44^{high}/CD24$_{low}$ markers of MCF10A H-Ras^{V12} cells cultured on soft agar or ultralow attachment plates. Analysis was performed 5 days after seeding. (G) Expression of mesenchymal markers *VIM, ZEB1, SLUG* and epithelial marker *E-CAD* in MCF10A H-Ras^{V12} cells cultured on soft agar or ultralow attachment plates. Analysis was performed 5 days after seeding. (H) Representative pictures of MCF10A and MCF10A H-Ras^{V12} cells cultured on soft-agar with 0.5% Matrigel. Analysis was performed at day 5. Scale bar: 150 µm. (I) Expression of mesenchymal markers *VIM, ZEB1, SLUG* and epithelial marker *E-CAD* in MCF10A cells cultured on soft-agar with 0.5% Matrigel (3D) or attached (2D) conditions measured by qRT-PCR. (J) Expression of mesenchymal markers *VIM, ZEB1, SLUG* and epithelial marker *E-CAD* measured in MCF10A H-Ras^{V12} cells cultured on soft-agar with 0.5% Matrigel (3D) or attached (2D) conditions by qRT-PCR. All error bars represent standard deviation (n≥3). Two-tailed unpaired student's T-test was performed. * denotes $p \leq 0.05$; ** denotes $p \leq 0.01$, *** denotes $p \leq 0.001$.

H-Ras^{V12} overexpression leads to an epithelial to mesenchymal transition (EMT) in MCF10A cells[1-3], therefore we decide to measure selected key genes of EMT to ensure we recapitulate the published phenotype of these cells and to be able to decide for the most suitable 3D cultivation system for our study. We tested two different 3D culture

conditions and found that cultivation on soft-agar (compared to ultralow attachment plates) resulted in increased colony homogeneity (less colony aggregation) and increased expression of markers linked to EMT in MCF10A H-Ras^{V12} cells (Supplementary Figure 1E-G). Next, we characterized the same features in MCF10A versus MCF10A H-Ras^{V12} cells cultured in 2D versus 3D conditions. Since non-transformed MCF10A require matrix attachment for 3D growth, we added 0.5% Matrigel to the culture medium. We found that 3D culture conditions intensify differences between MCF10A and MCF10A H-Ras^{V12} cells based on *VIM, ZEB1, SLUG* and *E-CAD* as well as the CD44^{high}/CD24$_{low}$ expression (Supplementary Figure 1 H-J).

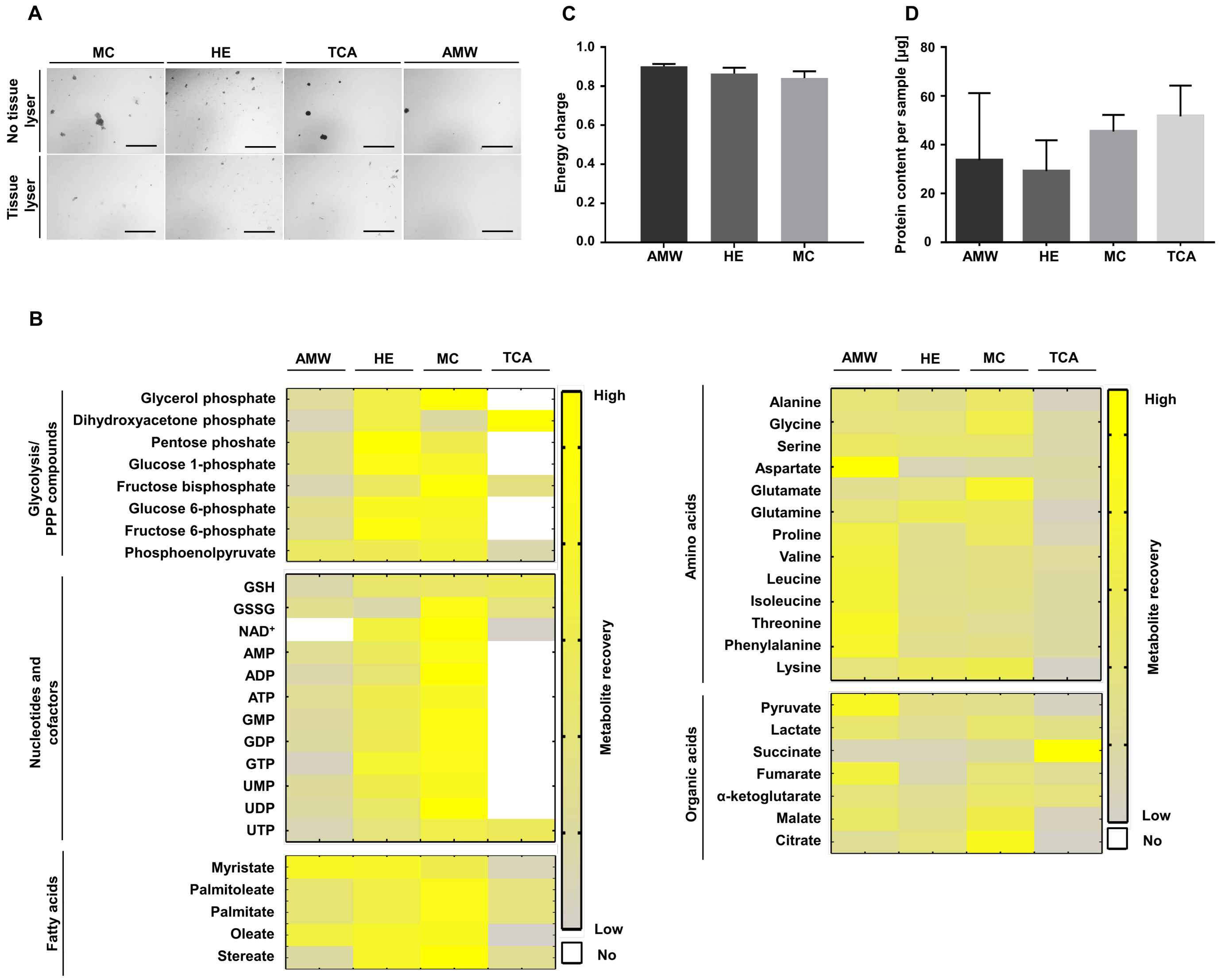

Supplementary Figure 2: Metabolite extraction based on methanol-chloroform combined with mechanical disruption is suitable for soft-agar (3D) cultures

Four different extraction methods were tested: AMW (Acetonitrile Methanol Water), HE (Hot Ethanol), MC (Methanol Chloroform), and TCA (Trichloroacetic acid). (A) Representative pictures of spheroids after the extraction procedures with or without mechanical disruption (tissue lyser). Scale bar: 300 µm. (B) Heat map representing metabolite recovery (extraction efficiency and metabolite preservation) across the different extraction methods based on soft-agar cultured MCF10A H-Ras^{V12} cells. White indicates that the metabolite was not detectable by the mass spectrometry analysis and thus not recovered by the extraction method. Gray indicates that the metabolite recovery was low, which means the metabolite peak intensity was below the average of the metabolite peak intensity across all extraction methods. Yellow indicates that the metabolite recovery was high, which means the metabolite peak intensity was above the average of the metabolite peak intensity across all extraction methods. Glycolysis/Pentose Phosphate Pathway (PPP) metabolites, nucleotides, and cofactors were measured with LC-MS, while amino acids, organic acids, and fatty acids were measured with GC-MS. (C) Energy charge determined from soft-agar cultured MCF10A H-Ras^{V12} cells. (D) Protein content determined from soft-agar cultured MCF10A H-Ras^{V12} cells using a BCA assay. All error bars represent standard deviation (n=3).

We tested which previously published extraction method[4-7] is most suited to extract metabolites from soft-agar (3D) cultures. We found that for each extraction method a mechanical disruption step with a tissue lyser was necessary for complete spheroid destruction (Supplementary Figure 2A). Next, we tested how many different intracellular metabolites we could detect based on the different chemical extraction methods combined with the mechanical disruption step. We found that MC compared to AMW, HE, and TCA extraction resulted in a higher metabolite

recovery across all chemical classes of metabolites and consequently resulted in an increased number of detected metabolites (Supplementary Figure 2B). Next, we determined the energy charge and the protein recovery based on the different chemical extraction methods combined with the mechanical disruption step. The energy charge was very similar between MC, AMW, and HE extraction (Supplementary Figure 2C), yet when comparing the protein recovery MC extractions yielded higher protein recovery and less variability within the replicates, presumably due to the biphasic nature of this extraction method (Supplementary Figure 2D). Thus, we concluded that methanol-chloroform (MC) combined with mechanical disruption is most suited for extracting metabolites from 3D soft-agar cultures.

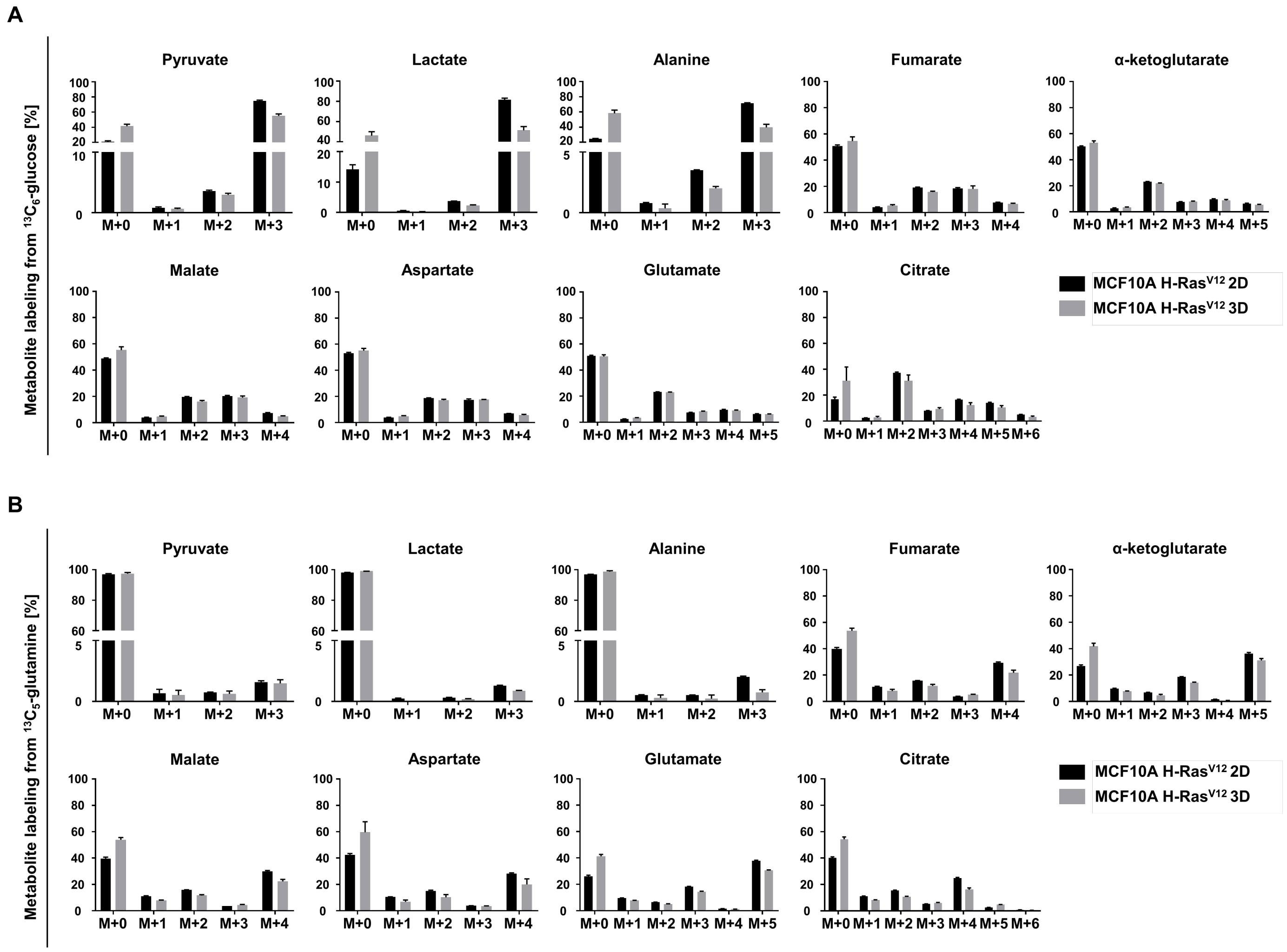

Supplementary Figure 3: Glucose and glutamine contribution to organic and amino acid production is altered in MCF10A H-Ras^{V12} spheroids (3D) compared to attached (2D) cells

Metabolite labeling from $^{13}C_6$-glucose (A) or $^{13}C_5$-glutamine (B) in MCF10A H-Ras^{V12} cells cultured on soft-agar (3D) or attached (2D) conditions. All error bars represent standard deviation (n=3).

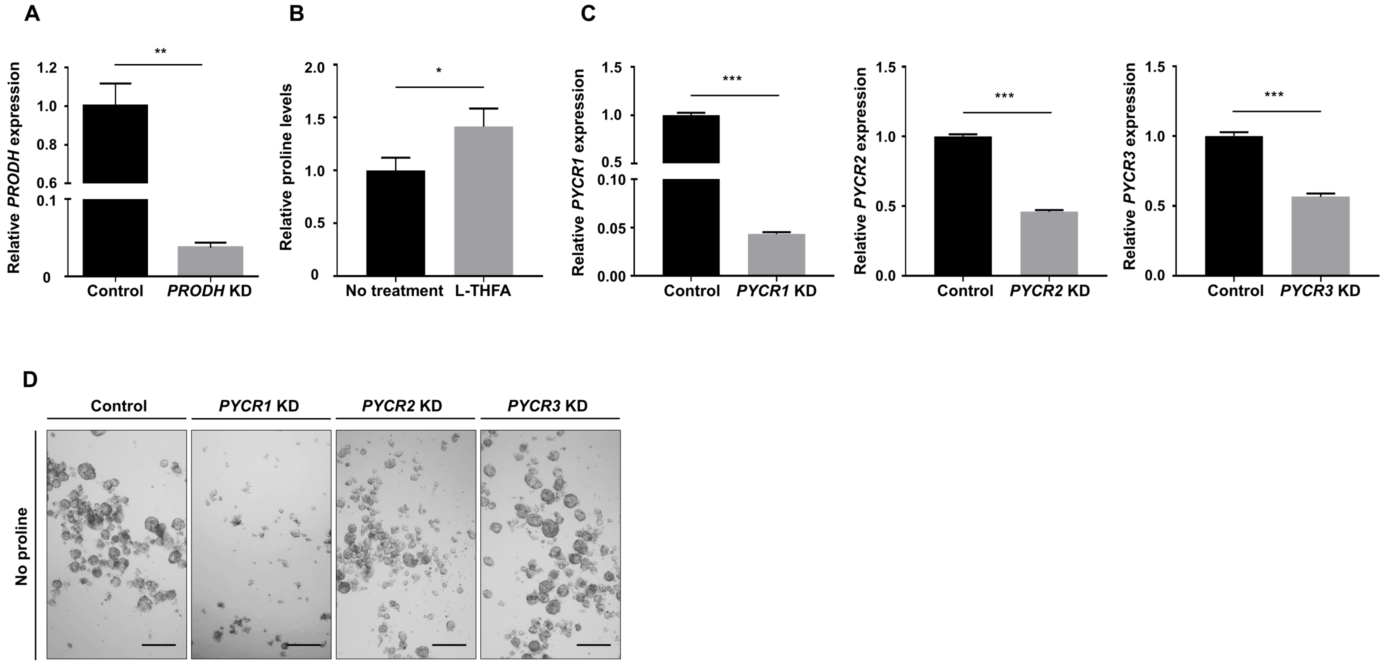

Supplementary Figure 4: Genetic and catalytic inhibition of proline metabolism in MCF10A H-Ras^{V12} spheroids (3D)
(A) *PRODH* expression of MCF10A H-Ras^{V12} cells with or without *PRODH* knockdown (KD). Knockdown was generated using iCRISPR transcriptional repression. (B) Intracellular levels of proline upon L-THFA treatment in MCF10A H-Ras^{V12} spheroids (3D). Treatment was started at day 0. Analysis was performed at day 5 of treatment. (C) *PYCR1,2,3* expression of MCF10A H-Ras^{V12} cells with or without the corresponding *PYCR* knockdown (KD). Knockdowns were generated using shRNA. (D) Representative pictures of MCF10A H-Ras^{V12} spheroids transduced with a lentiviral vector with shRNA for *PYCR1, PYCR2, PYCR3* (KD) or a scrambled sequence in media without proline. Analysis was performed at day 5. Scale bar: 150 µm. All error bars represent standard deviation (n=3). Two-tailed unpaired student's T-test was performed. * denotes p ≤ 0.05; ** denotes p ≤ 0.01, *** denotes p ≤ 0.001.

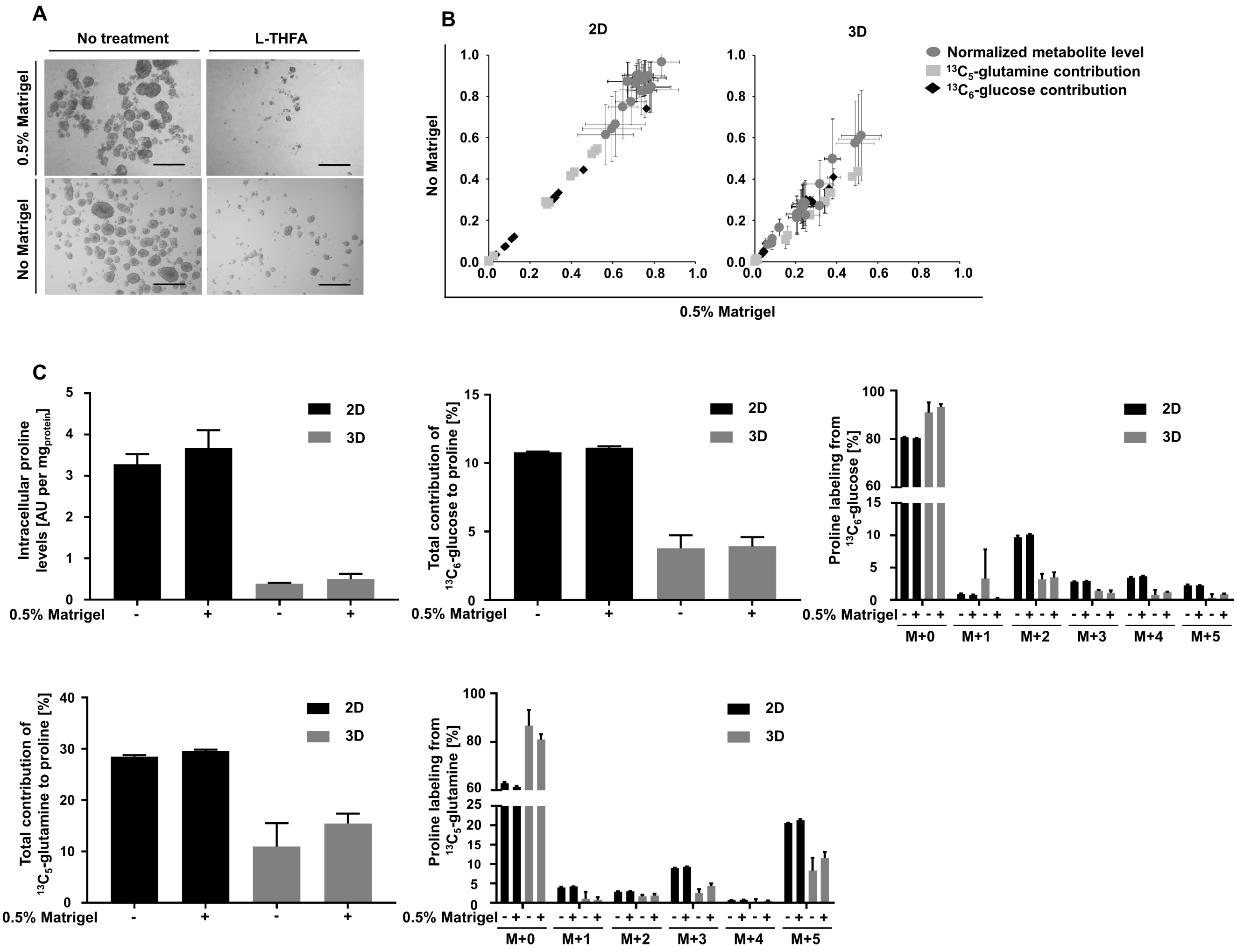

Supplementary Figure 5: Addition of 0.5% Matrigel to soft-agar cultures does not significantly alter TCA cycle and amino acid metabolism in MCF10A H-Ras^{V12} cells

(A) Representative pictures of MCF10A H-Ras^{V12} spheroids cultured in media with or without 0.5% Matrigel upon treatment with L-THFA compared to no treatment. Treatment was started at day 0. Analysis was performed at day 5. Scale bar: 150 µm. (B) Correlation of the total $^{13}C_6$-glucose and $^{13}C_5$-glutamine contribution as well as metabolite levels of MCF10A H-Ras^{V12} cells cultured in 2D and in 3D with or without 0.5% Matrigel. Metabolite levels were normalized for protein content and internal standard and scaled by the highest value of each group. (C) Intracellular proline levels, $^{13}C_6$-glucose and $^{13}C_5$-glutamine contribution to proline in MCF10A H-Ras^{V12} cells cultured in 2D and in 3D with or without 0.5% Matrigel. All error bars represent standard deviation (n=3). Two-tailed unpaired student's T-test was performed.

A

Hematology

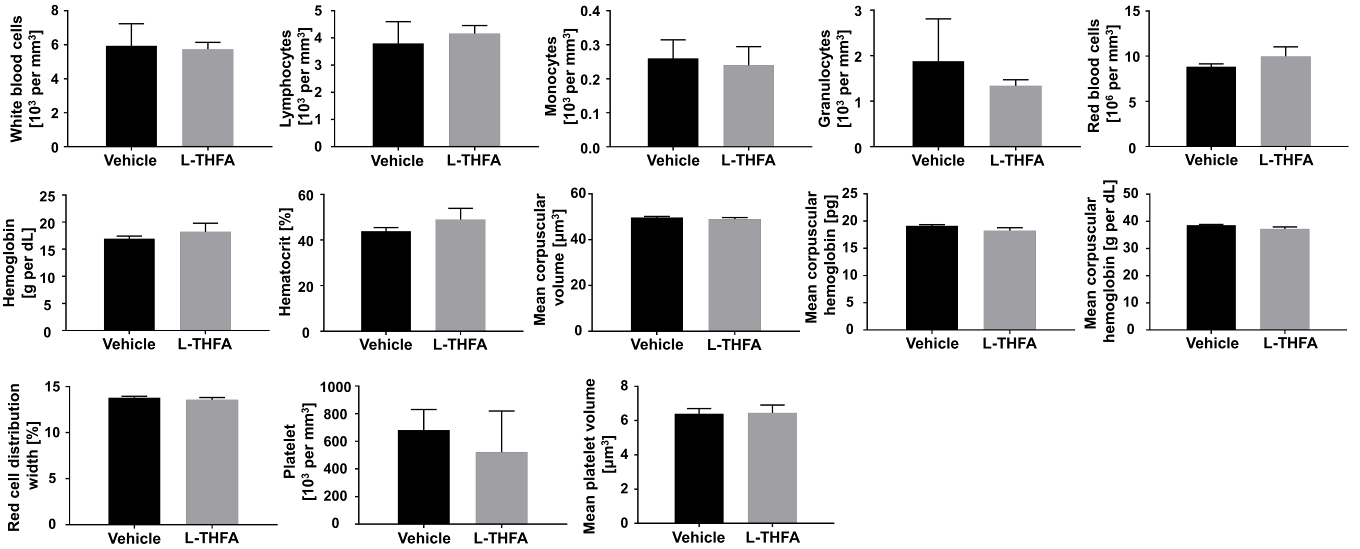

B

Organ weight

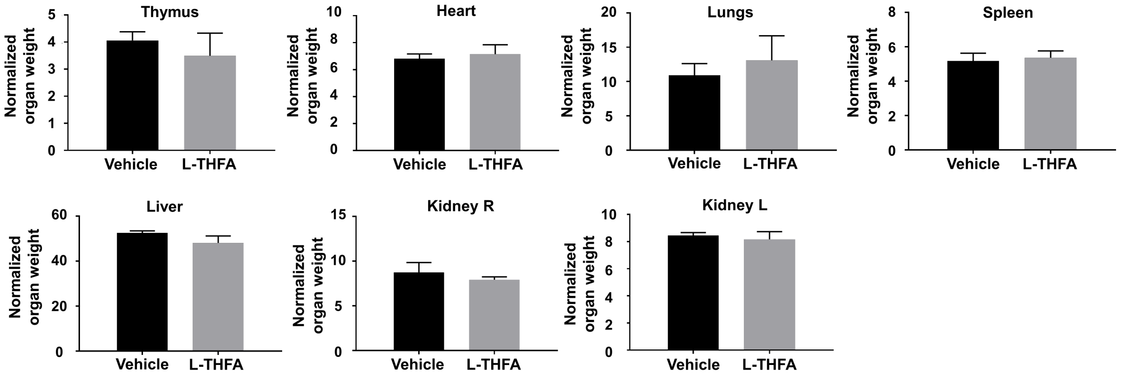

C

Analytical chemistry

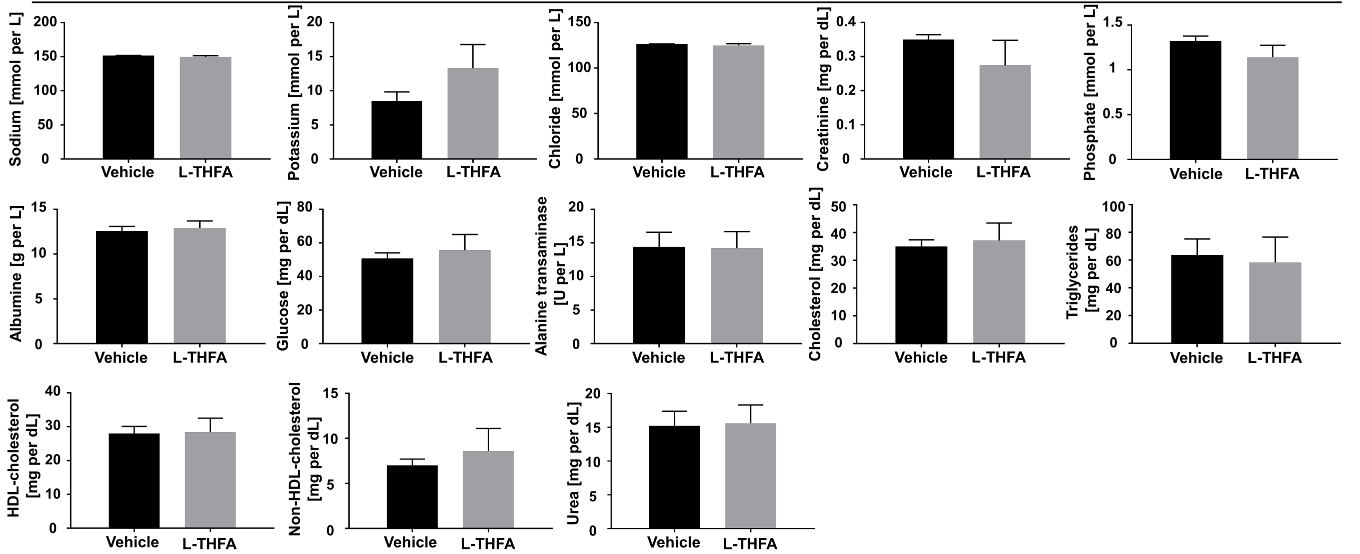

D

Histopathological examination

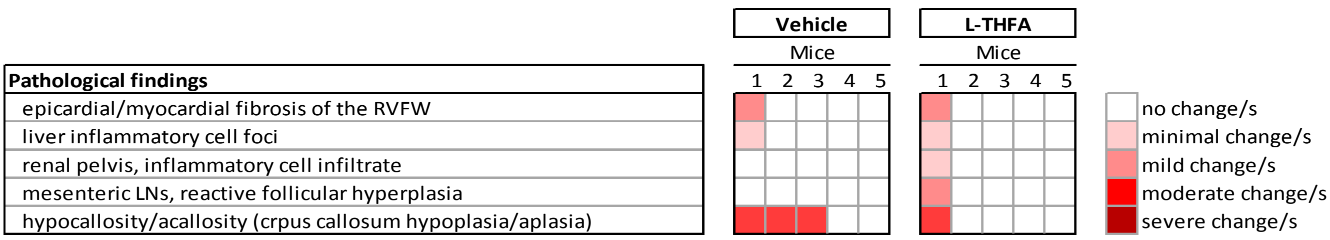

Supplementary Figure 6: L-THFA treatment does not have any obvious adverse effects

Hematology (A), organ weight (B), analytical chemistry (C), and histopathological examination (D) of BALB/c mice treated with vehicle (PBS) or L-THFA (30 mg per kg) for 16 days. Error bars represent standard deviation (n=5). Two-tailed student's T-test with F-testing to confirm equal variance was performed.

Gross examination at necropsy did not reveal any macroscopic change. Concerning organ weights, hematology and clinical chemistry, no statistically significant differences were observed among groups (Supplementary Figure 6A,B,C). The results of histopathological examination are summarized in Supplementary Figure 6D. Also in this case, significant variations have not been observed among the experimental groups in terms of lesion spectrum, frequency and severity. Most of the recorded lesions (*e.g.* inflammatory cell foci in different tissues, reactive follicular hyperplasia of lymph node) can be interpreted as spontaneous and not treatment-related findings that are often reported in sub-acute or chronic toxicologic studies. Hypocallosity/acallosity of the brain and epicardial/myocardial fibrosis of the right ventricular free wall represent strain-specific conditions that are commonly reported in BALB/c mice.

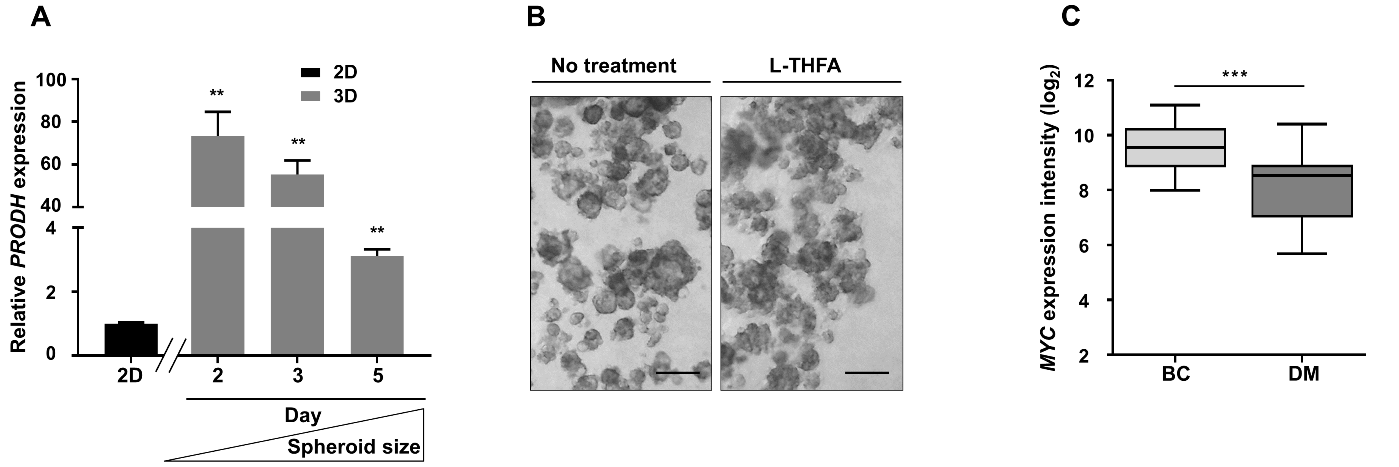

Supplementary Figure 7: *PRODH* expression inversely correlates with spheroid size and *MYC* expression

(A) Relative expression of *PRODH* in MCF10A H-Ras^{V12} cells spheroids (3D) at different days relative to MCF10A H-Ras^{V12} cells in 2D culture. With time spheroid size increases. Error bars represent standard deviation (n=3). Two-tailed unpaired student's T-test was performed. ** denotes $p \leq 0.01$. (B) Representative pictures of MCF10A H-Ras^{V12} spheroids upon treatment with L-THFA compared to no treatment. Treatment was started at day 3. Analysis was performed at day 5. Scale bar: 150 µm. (C) *MYC* expression levels in primary breast cancer (BC) tissue (GSE20711) and breast cancer-derived metastases (DM) tissue (GSE14017) from patients. Data are shown as medians (bar) with the 25th–75th percentile range (box) and 10th–90th percentile range (whiskers). Two-tailed unpaired student's T-test with Welch`s correction was performed. *** denotes $p \leq 0.001$.

Supplementary Table 1: shRNA sequences used to generate cell lines with enzyme knockdowns

Lentiviral pLKO	Sequence
shPYCR1	CCGGCACAGTTTCTGCTCTCAGGAACTCGAGTTCCTGAGAGCAGAAACTGTGTTTTTG
shPYCR2	CCGGGCCCTTAAGAAGACCCTCTTACTCGAGTAAGAGGGTCTTCTTAAGGGCTTTTTG
shPYCR3	CCGGATCCTAAGCTCCTGACAAGTGCTCGAGCACTTGTCAGGAGCTTAGGATTTTTT
Scrambled	ATCTCGCTTGGGCGAGAGTAAG

Supplementary Table 2: Primer sequences used to determine gene expression levels

Gene Abbreviation	Gene Name	Primer Sequence
RPL-19	Ribosomal protein L19	Fw: 5’ - attggtctcattggggtctaac - 3’<br>Rv: 5’ - agtatgctcaggcttcagaaga - 3’
VIM	Vimentin	Fw: 5’ - gaccagctaaccaacgacaaa - 3’<br>Rv: 5’ - tgaaagattgcagggtgttt - 3’
ZEB1	Zinc Finger E-Box Binding Homeobox 1	Fw: 5’ - agacatgtgacgcagtctgggt - 3’<br>Rv: 5’ - tgggcattcatatggcttctctcca - 3’
SLUG	Snail Family Zinc Finger 2	Fw: 5’ - cttcctggtcaagaagcatt - 3’<br>Rv: 5’ - tgaggagtatccggaaagag - 3’
E-CAD	E-Cadherin	Fw: 5’ - gaacgcattgccacatacac - 3’<br>Rv: 5’ - attcgggcttgttgtcattc - 3’
PRODH	Proline dehydrogenase	Fw: 5’ - tgaagctgtgtggctgaaac - 3’<br>Rv: 5’ - cgcagagaaaaggcaaactc - 3’
P5CDH	Delta-1-pyrroline-5-carboxylate dehydrogenase	Fw: 5’ - atggccactacccccttatcc - 3’<br>Rv: 5’ - ctttaccctggacacctgga - 3’
P5Cs	Delta-1-pyrroline-5-carboxylate synthase	Fw: 5’ - agtccccctttcgcatttagt - 3’<br>Rv: 5’ - aagctgcaagcatctggaat - 3’
PYCR1	Pyrroline-5-carboxylate reductase	Fw: 5’ - catctgctcattcacgcact - 3’<br>Rv: 5’ - aacctatgtggggagcacag - 3’
PYCR2	Pyrroline-5-carboxylate reductase 2	Fw: 5’ - gacgtcctgtttctggctgt- 3’<br>Rv: 5’ - ctccacagagctgatggtga - 3’
PYCR3	Pyrroline-5-carboxylate reductase 3	Fw: 5’ - cgtcatctttgccaccaag - 3’<br>Rv: 5’ - cacggacaccaagatgtgtt - 3’

Supplementary References:

1. Shin, S. & Blenis, J. ERK2/Fra1/ZEB pathway induces epithelial-to-mesenchymal transition. *Cell Cycle* **9**, 2483-2484 (2010).
2. Yoh, K.E. *et al.* Repression of p63 and induction of EMT by mutant Ras in mammary epithelial cells. *Proc Natl Acad Sci U S A* **113**, E6107-E6116 (2016).
3. Morel, A.P. *et al.* Generation of breast cancer stem cells through epithelial-mesenchymal transition. *PLoS One* **3**, e2888 (2008).
4. de Koning, W. & van Dam, K. A method for the determination of changes of glycolytic metabolites in yeast on a subsecond time scale using extraction at neutral pH. *Anal Biochem* **204**, 118-123 (1992).
5. Bent, K.J. & Morton, A.G. Amino acid composition of fungi during development in submerged culture. *Biochem J* **92**, 260-269 (1964).
6. Tunnicliffe, H.E. Glutathione: The Occurrence and Quantitative Estimation of Glutathione in Tissues. *Biochem J* **19**, 194-198 (1925).
7. Rabinowitz, J.D. & Kimball, E. Acidic acetonitrile for cellular metabolome extraction from Escherichia coli. *Anal Chem* **79**, 6167-6173 (2007).
